# Evaluation of tracking accuracy of the CyberKnife system using a webcam and printed calibrated grid

**DOI:** 10.1120/jacmp.v17i2.5914

**Published:** 2016-03-08

**Authors:** Iori Sumida, Hiroya Shiomi, Naokazu Higashinaka, Yoshikazu Murashima, Youichi Miyamoto, Hideya Yamazaki, Nobuhisa Mabuchi, Eimei Tsuda, Kazuhiko Ogawa

**Affiliations:** ^1^ Department of Radiation Oncology Osaka University Graduate School of Medicine 2‐2 Yamada‐oka, Suita Osaka 565‐0871 Japan; ^2^ CyberKnife Center Soseikai Clinic 126 Shimotobakamimisucho, Kyoto Fushimi‐ku Kyoto 612‐8248 Japan

**Keywords:** CyberKnife, synchrony, motion tracking, quality assurance, verification system

## Abstract

Tracking accuracy for the CyberKnife's Synchrony system is commonly evaluated using a film‐based verification method. We have evaluated a verification system that uses a webcam and a printed calibrated grid to verify tracking accuracy over three different motion patterns. A box with an attached printed calibrated grid and four fiducial markers was attached to the motion phantom. A target marker was positioned at the grid's center. The box was set up using the other three markers. Target tracking accuracy was evaluated under three conditions: 1) stationary; 2) sinusoidal motion with different amplitudes of 5, 10, 15, and 20 mm for the same cycle of 4 s and different cycles of 2, 4, 6, and 8 s with the same amplitude of 15 mm; and 3) irregular breathing patterns in six human volunteers breathing normally. Infrared markers were placed on the volunteers’ abdomens, and their trajectories were used to simulate the target motion. All tests were performed with one‐dimensional motion in craniocaudal direction. The webcam captured the grid's motion and a laser beam was used to simulate the CyberKnife's beam. Tracking error was defined as the difference between the grid's center and the laser beam. With a stationary target, mean tracking error was measured at 0.4 mm. For sinusoidal motion, tracking error was less than 2 mm for any amplitude and breathing cycle. For the volunteers’ breathing patterns, the mean tracking error range was 0.78‐1.67 mm. Therefore, accurate lesion targeting requires individual quality assurance for each patient.

PACS number(s): 87.55.D‐, 87.55.km, 87.55.Qr, 87.56.Fc

## I. INTRODUCTION

Respiratory motion varies substantially between patients,^(1)^ and the consequent tumor motion during radiotherapy can compromise the accuracy of dose delivery and produce interplay and blurring effects.^2^, ^3^ Thus, various methods have been devised to compensate for motion, including dynamic multileaf collimator (DMLC) tracking^(4)^ and the use of a robotic arm with six axes.^(5)^ The CyberKnife system (Accuray Inc., Sunnyvale, CA) uses image guidance with two pairs of X‐ray tubes and a flat‐panel detector to detect bone anatomy, fiducial marker position, and tumor position according to image densities.^(6)^ Although the overall system‐targeting precision is less than 1 mm^7^, ^8^ the accuracy of the robotic system is highly variable, as reflected by the precision of production and the efficacy of control.

The American Association of Physicists in Medicine Task Group 135 introduced tolerance values and quality assurance (QA) intervals for motion tracking using film.^(9)^ Accordingly, the company Accuray provided a QA phantom, which moves in a sinusoidal pattern, and Nioutsikou et al.^(10)^ evaluated the tracking accuracy of the CyberKnife system using film and gamma analyses during lung treatments. In addition, Chan et al.^(11)^ evaluated the accuracy and sensitivity of four‐dimensional dose calculations in the treatment planning system of CyberKnife, and made comparisons with measured film data from tracking irradiation. Although tracking system accuracy with film irradiation may be achievable as an overall tracking result, tracking errors of each beam remain difficult to determine. Hence, motion phantoms that can move in all directions to simulate tumor motion are used to predict motion‐tracking accuracy. Moreover, several previous studies report detection of irradiation targets motions using charge‐coupled device (CCD) cameras.^12^, ^13^ However, this method requires attachment of the camera to coaxial fixation jigs, which are not commercially available.^(13)^ Thus, to determine the precision of the CyberKnife system, Wong et al.^(14)^ attached LED markers to the CyberKnife head and to the target, and simultaneously evaluated precision during irradiation.

The aim of the present study was to evaluate tracking accuracy of the CyberKnife system using a webcam and a printed calibrated grid over various motion patterns, including those of the stationary position, sinusoidal motion, and human respiratory motion.

## II. MATERIALS AND METHODS

The CyberKnife system is a 6 MV linear accelerator that is mounted on a robotic arm. The system includes the Synchrony Respiratory Tracking System v.9.6, which makes real‐time adjustments to the robotic arm position to follow the moving target.^15^, ^16^, ^17^ The target locating system (TLS) is composed of two ceiling‐mounted diagnostic X‐ray sources paired with amorphous silicon detectors, and acquires live digital radiographic images of the target, bony anatomy, and fiducial markers. The Synchrony system enables three‐dimensional real‐time tracking of tumors that move with respiration.^(18)^ The system uses infrared markers that are continually tracked by three flash‐point cameras, which generate 26 images per second. The TLS acquires two orthogonal X‐ray images within a preset interval to locate three‐dimensional positions of fiducial markers, which are implanted into, or are present close to, the tumor. The Synchrony system then creates correlation models between positions of external infrared markers and fiducial markers, and tracks the tumor according to predictions that are based on infrared marker positions. This system allows tumors that are subject to respiratory motion to be treated with an accuracy of 2 mm or less during normal respiration.^19^, ^20^

### A. Phantom configuration

**Figure 1:**
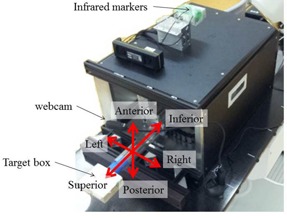
Motion phantom movable in all directions. A webcam on a flexible arm was placed on the treatment couch and the target box was attached to the end of the motion phantom.

**Figure 2:**
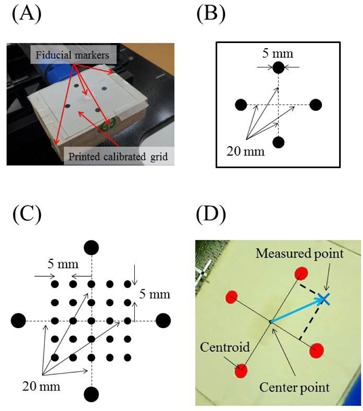
A printed calibrated grid on the target box (a). Four fiducial markers were attached to the box on the top, the two sides, and in the center of four black solid circles. (b) Four black solid circles were printed on white paper. Circles of 5 mm in diameter were placed 40 mm from the other circle centers. (c) A printed calibrated grid was used to verify coordinate recognition accuracy. Four large solid black circles and 25 small solid black circles were printed on a sheet. The four large circles were used to create the coordinate system on the sheet. The 25 small circles were used to verify detection accuracy. (d) Four solid red circles were detected by the software.

We used the motion phantom from Enomoto BeA Co., Ltd. (Gifu, Japan), which simulates respiratory motion by moving in axial, sagittal, and coronal planes, and in oblique axes (Fig. 1).^(21)^ Infrared markers were placed on the phantom, and a webcam (BSW20KM14BK, Buffalo Inc., Aichi, Japan) was used to capture video signals in AVI file format at 30 frames/s. The webcam produces a pixel matrix of 720×480, and was placed on the treatment couch to afford a field of view that was unobstructed by the CyberKnife instrument. The target comprised a box covered with a printed calibrated grid, and was attached to the end of the motion phantom. The box had four fiducial markers (Fig. 2(a)), three of which were used to align the motion phantom with translations and rotations, which were detected using the fiducial tracking method in the Synchrony system. The tolerance for alignment of the motion phantom was 0.1 mm for translation and 0.1° for rotation. The other fiducial marker was used as a target.

### B. Validation of coordinate recognition accuracy

The software was developed using Delphi2007 (Borland Software Corporation, Austin, TX) to analyze tracking accuracy and to measure the detection accuracy of printed markers on the calibrated grid. Four marker points that were identical to the outer circles in Fig. 2(b) surrounded 25 smaller calibration points on the sheet (Fig. 2(c)), and were evenly spaced with 5 mm intervals. Centroids for each of the four black solid circles were calculated in pixels on the captured image using the software, and distances from the centroid of each of the four black solid circles to the center point were calculated in pixels. The center point was defined as the origin of the coordinate system (Fig. 2(d)). Distance in mm was divided by distance in pixels, giving a pixel size of 0.24. The centroids of the four black solid circles were considered vectors from the center point, and locations were calculated for all 25 marker points. Distances between these and expected locations were recorded as software calibration errors.

### C. Treatment planning using the fiducial tracking method

Planning CT scans of the motion phantom were acquired with a slice thickness of 1.25 mm, and phantom CT images were imported into the treatment planning system (TPS; Multiplan 4.6.0, Accuray, Inc., Sunnyvale, CA) via DICOM. The four fiducial markers were then delineated using the TPS, the beam isocenter was localized at the base of the designated fiducial marker, and an isocentric irradiation plan was devised. To record tracking using the webcam, irradiation beams to the target from the anterior direction for irradiation were chosen in the TPS. The vertical position of the source was less than 800 mm in accordance with the International Electrotechnical Commission coordinate system. The red laser was used as a substitute for the actual beam, and was hence available to visualize the red laser and the printed calibrated grid from the webcam. Incidence angles from the beam to the target ranged between 31° and 54°. A total of 20 fields were used and planned beams at the time of each field were changed to 240 monitor units (MUs) to maximize tracking times.

### D. Checking tracking accuracy for different breathing patterns

**Figure 3:**
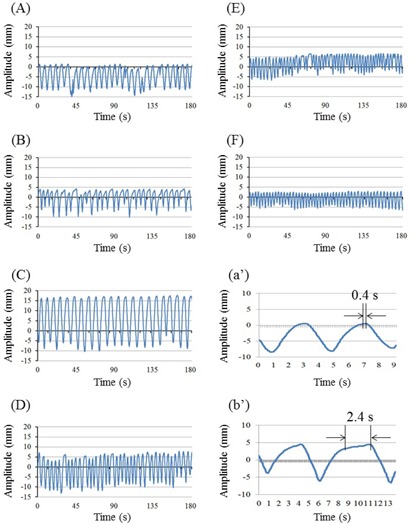
Breathing patterns of the six volunteers (A‐F). The y‐axis represents the breathing motion amplitude in millimeters. The expiratory and inspiratory phases increase and decrease Y coordinates, respectively. The x‐axis represents the recording time in seconds. Cases (A‐F) correspond with respective cases in Table 1, and (a’) and (b’) depict detailed patterns of (A) and (B), respectively, over two cycles. The distance between the two black arrows is a motionless pause; (a’) negligible pause between phases; (b’) measurable pause between phases.

Baseline data for tracking accuracy were obtained in the motionless state. Subsequently, sinusoidal motion patterns were evaluated in the superior‐inferior (SI) direction, and were assessed at amplitudes of 5, 10, 15, and 20 mm over 4 s with cycles of 2, 4, 6, and 8 s at an amplitude of 15 mm. Subsequently, six volunteers from cases (A) to (F) were recruited under appropriate institutional guidance from our department for evaluations of tracking accuracy during normal breathing. It was not possible to capture target motion using implanted fiducial markers because the volunteers were not patients. Thus, infrared markers were positioned on volunteers’ abdomens and positions were recorded using the Synchrony system. The largest degree of motion of the infrared marker in the anterior‐posterior (AP) direction was recorded and tumor trajectory in the SI direction was assumed equal. Hence, tumor trajectories were not identical to real tumor motion. Similarly, to estimate the abdominal motion of the phantom, the trajectory in the SI direction was also assumed in the AP direction, and the infrared markers moved with the tumor. Volunteers’ breathing patterns (Fig. 3) were recorded for approximately 3 min. Although the CyberKnife system performs motion tracking during longer treatment times, warranting the use of longer wave forms, these may limit the motion trajectory for the actual treatment. Accordingly, the motion of the phantom was limited and data for the upper motion limit were imported. Mean amplitudes and cycles of breathing patterns were recorded for all volunteers (Table 1).

The center point of the four solid black coordinate markers (Fig. 2(d)) was used as the target, and the CyberKnife red laser beam was used to represent the radiation beam center. The coincidence of laser beam and radiation beam centers in monthly QA tests was 0.4±0.1 mm (n=11). To calculate tracking accuracy, deviations between the red laser point and the center point were calculated by the software. These measurements were performed for stationary, sinusoidal motion, and breathing patterns of the six volunteers. Tracking errors were recorded at 16 frames/s in log files in text format. During detection of the red laser point by the software, the laser remained on for 1 to 2 s after the actual beam was turned off. Thus, to avoid false detection of tracking errors in motion cases, errors for the last 2 s of each beam were excluded from analyses. Cumulative histograms of the tracking errors of the motion pattern were calculated using log file data, and tracking errors were subdivided into magnitudes of 1, 2, 3, 4, and 5 mm and were presented in histograms.

**Table 1:** Mean amplitudes and breathing cycles (mean±1 SD) of six volunteer cases

	*Amplitude (mm)*	*Breathing cycle (s)*
*Case*	Mean±SD	Mean±SD
A	13.11±1.85	6.9±1.6
B	11.75±2.18	6.1±1.3
C	25.87±1.20	8.2±0.9
D	15.57±1.72	4.7±0.8
E	9.58±1.25	3.7±1.1
F	7.38±0.82	3.5±0.4

## III. RESULTS

### A. Validation of coordinate recognition accuracy by the software

The software located the 25 points on the printed calibrated grid and coordinate recognition accuracy was verified. When a region of 40×40 mm was defined to evaluate tracking errors, deviations between the expected point and the measured point were ≤0.33 mm (mean±SD, 0.13±0.09 mm), which was sufficient to evaluate tracking errors with the present software. Maximum deviations occurred at the four corners.

### B. Tracking accuracy: stationary

**Figure 4:**
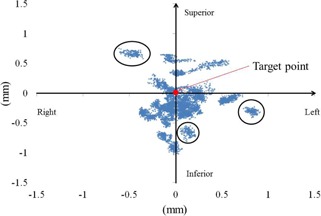
Laser point distributions around the target point for a stationary case. Blue marks represent laser points detected by the software. The target is shown as a solid red circle. Three black circles present laser point distributions at a certain beam. Although the robot aimed at the target point, deviations of distance were increased with incident beam angles.

In these experiments, laser points were aimed at a stationary target (Fig. 4) and tracking errors in left‐right (LR) and SI directions were determined using the software. These increased with decreasing angles of incidence of the laser beam to the target plane, and mean±standard deviations of LR and SI errors were 0.07±0.27 mm and −0.15±0.33 mm, respectively. The mean radial error was 0.39±0.23 mm.

### C. Tracking accuracy: sinusoidal motion

Tracking errors were measured during various breathing patterns against the intended data, and root mean square errors (RMSEs) were calculated. RMSEs were 0.49, 0.58, 0.67, and 0.76 mm for amplitudes of 5, 10, 15, and 20 mm, respectively, and were 1.01, 0.63, 0.53, and 0.49 mm for 2, 4, 6, and 8 s cycles, respectively. Magnitudes of tracking errors were correlated positively with amplitude (p<0.001) and were negatively correlated with cycle lengths (p<0.001).

**Figure 5:**
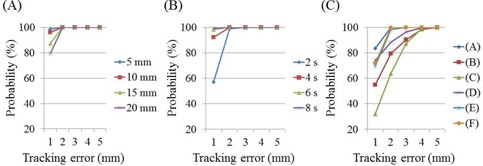
Probabilities of webcam‐detected tracking errors at different amplitudes (a), cycles (b), and in six volunteers (c).

Probabilities of irradiation for various tracking errors are shown in Figs. 5(a) and (b) using a cumulative distribution function (CDF). Probability was defined as the ratio of the time taken by the CyberKnife to track the target point with 1 to 5 mm tracking errors against the total tracking time×100. The time was calculated as the number of frames divided by 16 frames/s.

### D. Tracking accuracy: breathing patterns of six volunteers

RMSEs of webcam‐detected tracking errors in breathing patterns were 0.73, 1.62, 1.96, 1.20, 0.93, and 0.86 mm for cases (A), (B), (C), (D), (E), and (F), respectively. Probabilities of tracking errors (Fig. 5(c)) were calculated according to a CDF, and CyberKnife tracked the target point with errors of <2 mm with probabilities of 99.9%, 98.3%, and 99.8% for cases (A), (E), and (F), respectively. This probability gradually increased in the other volunteers over the tracking error range of 2‐5 mm, and was >95% for tracking errors of 4 mm in all cases.

## IV. DISCUSSION

According to the manufacturer,^(19)^ the tracking error of the CyberKnife using the Synchrony system during respiratory motion is less than 1.5 mm. However, this was measured using a vendor phantom that moves in a sinusoidal pattern, and breathing patterns of patients are generally not sinusoidal. Accordingly, Lujan et al.^(20)^ proposed a formula with an adjustable trajectory profile to better mimic patient breathing. Although the formula can be used to simulate patient breathing on the motion phantom, we assessed real breathing patterns and evaluated tracking accuracy under in situ conditions. To determine tracking accuracy of CyberKnife during irradiation with attention to time resolution, video webcam‐based analyses were performed. Inoue et al.^(13)^ detected tracking errors using a coaxial CCD camera attached to the beam exit. The CyberKnife tracks a colored ball that is controlled by the motion phantom in three dimensions and, when motion tracking is successful, the ball appears motionless on the video of the CCD camera. The present webcam acquires 30 frames/s with a 720×480 field of view. Although pixel‐scaling factors for the four directions depend on the field of view, 0.24 mm / pixel was achieved and was adequate for validation of coordinate recognition accuracy by the software. Pixel‐scaling factors increased with the distance of the measured point from the center. The same pixel scaling factor value was used in each axis and the required 0.3 mm / pixel tolerance was achieved. Although the printed calibrated grid was used for QA of tracking errors, use of the grid on volunteer abdomens may lead to deformations and rotations of the grid. However, upon detection of the four black solid circles, the software could calibrate according to the coordination of the grid during detection without changing the configuration of the printed calibrated grid, warranting further investigation in future studies.

In the present study, only 20 beams were used, whereas CyberKnife has 120‐140 beams for use in actual treatments. Thus, the overall accuracy of the CyberKnife tracking system cannot be assessed, and tracking accuracy requires further investigation over longer times. Nonetheless, the beam‐on time with 240 MUs irradiated by 800 MU / min was 18 s for each beam in the present study, and covered the longest breathing cycle length of 8.2 s, case (C) (Table 1).

In experiments with stationary targets, the mean tracking error using the fiducial tracking method was approximately 0.4 mm. However, errors increased with decreasing incident angles of the laser beam to the plane of the target. Incidence angles were calculated using the plan file in XML format, which describes source and target positions of each beam, and ranged between 31° and 54°. In light of the 0.3 mm targeting error in the AP direction, the incidence angle of 31° led to a tracking error of 0.5 mm. In contrast, Inoue et al.^(13)^ reported no influence of the incidence angle of each beam on the detection method, because the target shape was a circle. Moreover, Antypas et al.^(22)^ reported a motionless tracking accuracy of 0.19 mm using a film method, whereas the tracking error in the present detection method was approximately 0.4 mm. Hence, tracking errors may be reduced with the distance between the webcam and the plane of target, resulting in higher resolution analyses. Under conditions of larger numbers of beam directions and different DeltaMan settings, CyberKnife can only be calibrated to an accuracy of 0.5 mm. However, this error is strongly influenced by slice thicknesses of the phantoms for end‐to‐end test, even in the presence of coincident laser and actual beams, and by the accuracy of fiducial markers in the TPS. Hence, the present system inaccuracy of ≤0.5 mm is less than that shown in previous measurements. Moreover, according to Ho et al.,^(23)^ CT slice thicknesses in the range of 1‐1.5 mm did not affect tracking accuracy, and we used a slice thickness of 1.25 mm. Nonetheless, because thickness affects the resolution of digitally reconstructed radiography for assessments of registration accuracy, further studies using finer slice thicknesses are planned.

To verify tracking errors of moving targets, all tests of sinusoidal and irregular motions were performed according to one‐dimensional motions in the SI direction. We assumed that the motion of a lung tumor occurs in this direction, and the infrared marker on the abdomen was used to estimate tumor motion. Because the major axis of tumor motion inside the patient is in the SI direction rather than in AP or LR directions, the synchrony system reduces tracking errors.^(16)^ However, directions and amplitudes of the motion of normal tumors in the lung or liver differ in absolute terms from those of the chest and abdomen. Thus, although SI motions are sufficient for initial evaluations, future real tumor motion assessments from log files or from fluoroscopy analyses will be required to estimate 3D motions. During tracking of sinusoidal motions, tracking errors gradually increased with the amplitude of the same cycle, and with shorter cycles of the same amplitude. Thus, faster target movements gave greater tracking errors. Nonetheless, the Synchrony system tracked the target within a tracking error of 2 mm, and this was consistent with a previous study,^(9)^ but was greater than that reported by Inoue et al.,^(13)^ who demonstrated a median tracking error of 1 mm with a probability of >95% for 10 beams.

In the present study, we assessed breathing patterns in six volunteers over approximately 3 minutes. Because the Synchrony system can be used to track tumors during irradiation, evaluations of tracking errors are required for extended times. Accordingly, Ernst et al.^24^, ^25^ evaluated respiratory motion traces for an average duration of 71 min. However, previous assumptions of periodic breathing motions have not necessarily included variations in breathing patterns, such as different amplitudes and cycles.^22^, ^26^, ^27^ In this study, we generated a correlation model between fiducial and infrared markers, but did not update the model. Malinowski et al.^(28))^ suggested that the mean time to alarm for the CyberKnife Synchrony system was 1.1 min without updating the prediction model, and showed increasing model errors over time for the tumor‐surrogate relationship. However, this effect was abolished by updating the model during the treatment fraction.^(29)^ Because patient breathing motion can change completely over 10 to 15 min,^(30)^ future studies with real patient data should be performed with longer treatment times and with model updates. In actual treatments, the placement of infrared markers on the abdominal surface is critical for accurate correlations between external infrared markers and internal fiducial markers,^(31)^ and phase shifts are an important consideration.^(32)^ In the present analyses, we assumed that there were no phase shifts between external and internal markers in sinusoidal and irregular motion cases. Thus, further studies are required to evaluate changes in tracking accuracy with phase shifts using this verification system. However, assuming the absence of such phase shifts, tracking errors and standard deviations varied among the present six volunteers, with mean differences of 0.64‐1.67 mm. Thus, the Synchrony system tracked the target with an accuracy of over 95% within a tracking error tolerance of 4 mm. Inoue et al.^(13)^ showed that median tracking errors with a probability of >95% ranged between 1.0 and 3.5 mm. Similarly, the present tracking errors were between 1.4 and 3.7 mm.

The typical breathing patterns presented in Figs. 3(a') and (b') correspond to (a) and (b) in Fig. 3, but show more details of the two respiratory cycles. Specifically, in the absence of a pause between expiration and inspiration (Fig. 3(a’)), CyberKnife tracked the target accurately on the basis of the prediction model. However, in the presence of an appreciable pause between the end of expiration and beginning of inspiration and no motion of the target (Fig. 3(b’)), CyberKnife predictions were inaccurate and future target positions lost accuracy. Hence, according to investigations of prediction latency by Ernst et al.,^(25)^ the prediction models require further modification.

**Figure 6:**
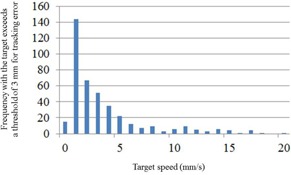
Correlation histograms between target speeds and frequencies of tracking errors for case (B). The vertical axis indicates the frequency with which the target exceeds a tracking‐error threshold of 3 mm.

Figures 3(b) and (c) show the same breathing patterns, potentially reflecting the large tracking errors of over 3 mm at the end of the expiratory phase in cases (B) and (C). Thus, we examined correlations between target speed and the frequency with which the target exceeded a threshold of 3 mm in case (B) (Fig. 6). These data were sampled and calculations were made under assumptions of normal distribution because the duration of tracking and the sampling frequency were sufficiently large, with target speeds of 0‐1 mm/s.

Estimating appropriate tracking accuracy requires performance of QA on a patient‐by‐patient basis. Specifically, pauses between the end of expiration and the beginning of inspiration could be eliminated by breath coaching, thereby reducing tracking error.

The present study was limited by isocentric performance of the irradiation technique and two‐dimensional assessments of tracking accuracy. Because the Synchrony tracking system can track tumors in three dimensions, the proposed webcam system should be considered in all three translational directions. In particular, the AP direction of the tumor has not been evaluated due to difficulty detecting changes in sizes of the four solid black circles, which would be used to calculate the magnitude of movements in the AP direction. Therefore, the webcam system requires further improvement, and use of a real radiation beam instead of the laser in combination with three‐dimensional dosimeters, such as plastic scintillation detectors or plenoptic cameras, would be useful.^(33)^ Moreover, the CyberKnife system commonly accommodates nonspherical tumors with nonisocentric beams from a semispherical space. Therefore, the verification system needs to include three‐dimensional tracking accuracy and adaptations for nonisocentric techniques.

## V. CONCLUSIONS

We evaluated a beam‐by‐beam verification system for tracking errors of the CyberKnife Synchrony system using a webcam and a printed calibrated grid, and calculated an overall treatment accuracy of 1‐2 mm for sinusoidal beam motions at various amplitudes and cycle lengths. However, varied median tracking errors for the six volunteers indicate the necessity of patient‐specific treatment margins.

## COPYRIGHT

This work is licensed under a Creative Commons Attribution 4.0 International License.

## Supporting information

Supplementary Material Files

Supplementary Material Files

Supplementary Material Files

Supplementary Material Files
