# Supplementary figures and images for: Evaluation of tracking accuracy of the CyberKnife system using a webcam and printed calibrated grid
Source: J Appl Clin Med Phys. 2016 Mar 8;17(2):74–84. doi: 10.1120/jacmp.v17i2.5914 (PMC5875552; doi:10.1120/jacmp.v17i2.5914)

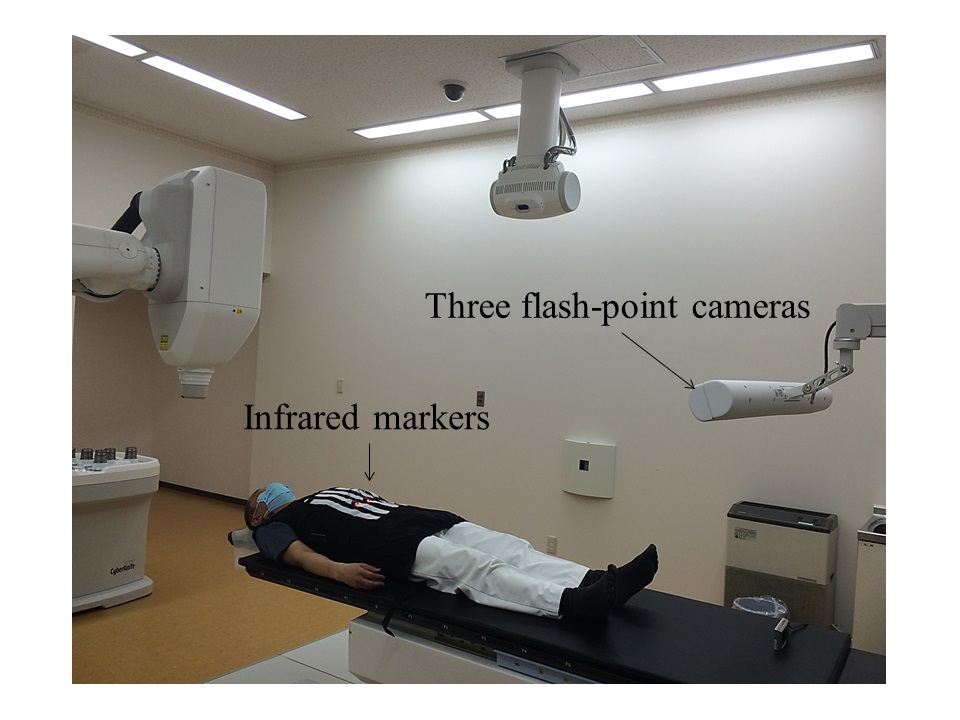

Supplement: Supplementary file 1 — Supplementary Material Files [file ACM2-17-74-s001.jpg]

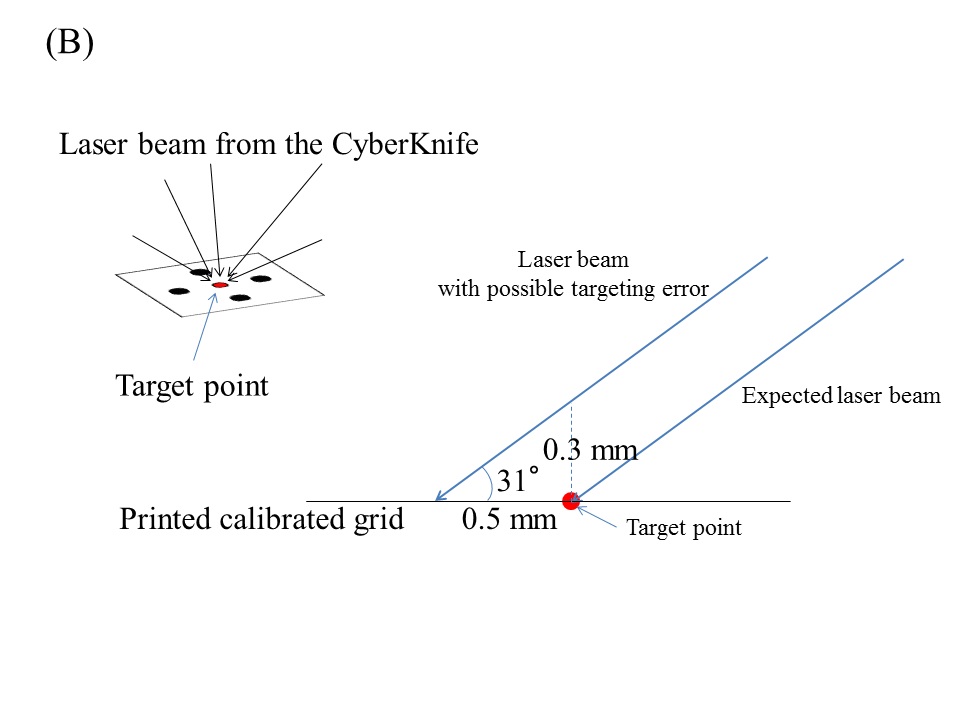

Supplement: Supplementary file 2 — Supplementary Material Files [file ACM2-17-74-s002.jpg]

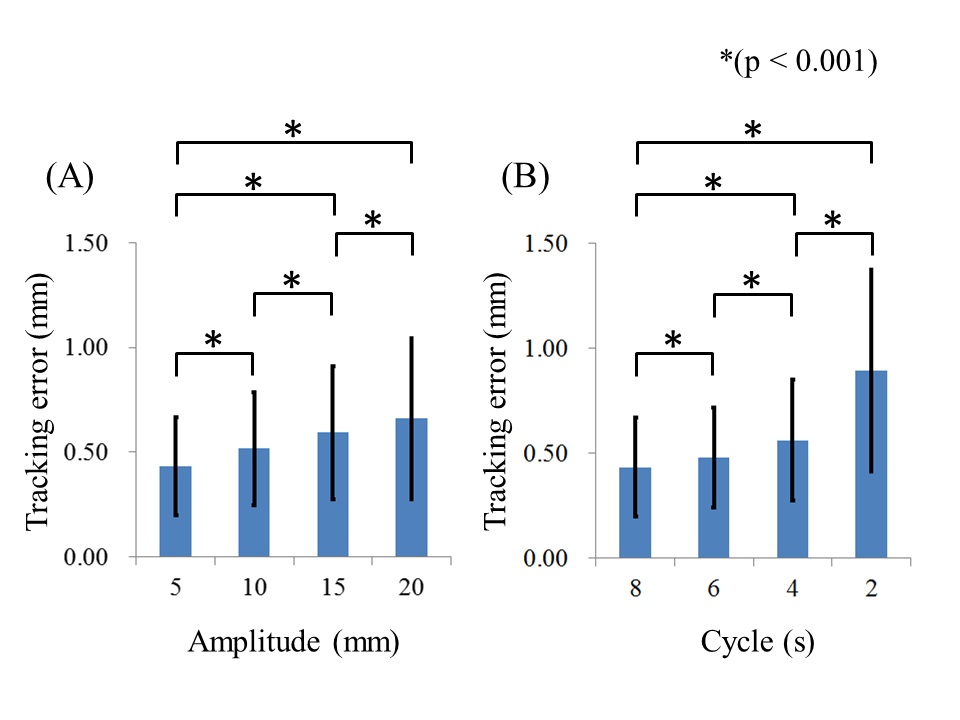

Supplement: Supplementary file 3 — Supplementary Material Files [file ACM2-17-74-s003.jpg]

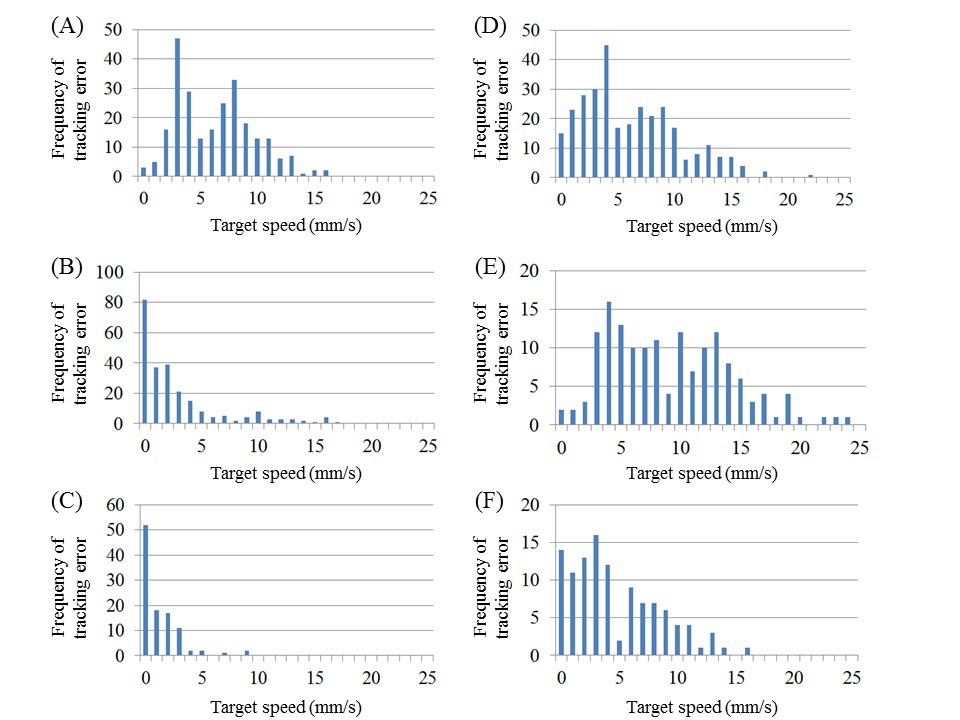

Supplement: Supplementary file 4 — Supplementary Material Files [file ACM2-17-74-s004.jpg]
